# Effects of Cigarette Smoking on Cardiac Autonomic Responses: A Cross-Sectional Study

**DOI:** 10.3390/ijerph17228571

**Published:** 2020-11-19

**Authors:** Nadeen Makhoul, Ishay Avivi, Sapir Barak Lanciano, Ella Haber Kaptsenel, Hana Bishara, Hagar Palacci, Chen Chaiat, Giris Jacob, Udi Nussinovitch

**Affiliations:** 1 Sackler Faculty of Medicine, Tel Aviv University, Tel Aviv 6997801, Israel; nadeen.makhoul@gmail.com (N.M.); ishayavivi@mail.tau.ac.il (I.A.); sapirbarak01@gmail.com (S.B.L.); ellakap2590@gmail.com (E.H.K.); hana.bishara@gmail.com (H.B.); hagar10194@gmail.com (H.P.); chaiatchen@gmail.com (C.C.); jacobgi@tlvmc.gov.il (G.J.); 2 Departments of Medicine F and J, Recanati Autonomic Dysfunction Center, Tel Aviv Sourasky Medical Center, Tel Aviv 6423906, Israel; 3 Department of Cardiology and Applicative Cardiovascular Research Center (ACRC), Meir Medical Center, Kfar Saba 4428164, Israel

**Keywords:** cigarette smoking, nicotine, autonomic nervous system, heart rate variability, deep breathing test

## Abstract

It has been suggested that some of the adverse, long-term cardiovascular outcomes of smoking are mediated by impaired autonomic nervous system (ANS) activity. Yet, this association is currently inconclusive. Heart rate variability (HRV) and the deep breathing test (DBT) represent common quantitative markers of ANS activity due to their simplicity and reliability. This large cross-sectional study was designed to assess the effect of active smoking on ANS function as manifested by HRV or DBT abnormalities. Electrocardiograms were recorded at rest for 5 min and during forced metronomic breathing. HRV and DBT were calculated according to accepted standards. Participants were divided into two groups based on current smoking status. The study included 242 healthy volunteers (196 nonsmokers and 46 smokers). There were no significant differences in age, sex, and BMI between groups. Cumulative smoking exposure burden (CSEB) for the study group was 5.3 ± 1.3 pack-years. Comparative analysis of HRV and DBT parameters according to smoking status revealed no significant differences between groups. Significant (*p* < 0.05), yet weak or moderate correlations (r < 0.7) were found between CSEB and abnormal change in HRV parameters consistent with sympathetic overactivity and decreased parasympathetic tone. In conclusion, smoking for a relatively short period in healthy adults does not seem to lead to significant impairment in ANS function. Yet, the consequences of smoking seem to be amplified when cumulative exposure burden increases.

## 1. Introduction

Several studies reported a significant relationship between autonomic nervous system (ANS) dysfunction and cardiovascular mortality, including sudden cardiac death [1,2,3]. As a result, efforts were spurred to develop various diagnostic tests to demonstrate the association between propensity for lethal arrhythmias and signs of increased sympathetic or reduced vagal activity in smokers [4].

Heart rate variability (HRV) is a well-established clinical tool for ANS evaluating function. This straightforward test is correlated with cardiovascular prognosis. Although HRV provides valuable data on ANS function at rest, it does not reflect autonomic response to stimuli, which entails additional tests, such as heart rate response metronomic deep breathing [5]. Thus, an additional diagnostic test, the deep breathing test (DBT), which differs from HRV, adds valuable physiologic information by quantitatively evaluating heart rate changes in response to autonomic stimuli normally associated with decreased vagal components [6].

Chronic cigarette smoking generates several devastating health effects, including increased risk for cardiovascular morbidity and mortality. Despite increasing awareness of these effects, it continues to be common, globally. It was suggested that cigarette smoking appears to acutely initiate cardiac autonomic imbalance [7,8,9]. Whereas the short-term effects on ANS dysfunction may be transient, the cumulative effects remain less clear [10]. Several studies suggest that chronic, active smoking generates marked disruptions in ANS function and, thus, increases the tendency for the development of cardiac arrhythmias. Specifically, smoking was suggested to increase sympathetic drive and reduce parasympathetic tone [7,11,12], although other studies reported conflicting results [10,13,14]. Diminished vagal control, as manifested by abnormal DBT responses, has been suggested as a possible mechanism linking smoking to cardiovascular deaths [7,15] and to increased mortality after acute myocardial infarction [16]. However, the value of DBT in evaluating cardiac autonomic control in active smokers has not been sufficiently studied.

This study was conceived to investigate the effects of active chronic cigarette smoking on cardiac ANS dysfunction in a large, cross-sectional cohort of volunteers with no known cardiovascular disease. Both HRV test and DBT were used in an attempt to settle the debate regarding possible associations between smoking and ANS dysfunction.

## 2. Materials and Methods

### 2.1. Study Design

A cross-sectional design was used. The institutional review board approved the research protocol, and all participants provided written informed consent.

### 2.2. Study Population

Volunteers were recruited from among Meir Medical Cente’s employees and their family members. Exclusion criteria were younger than 18 years of age, pregnancy, known cardiovascular disease, diabetes, or hypertension as well as the use of any drug, inhaler, or ingestible medication. Mild dyslipidemia for which life style modification was advised and familial history of hypertension or diabetes were not considered exclusion criteria. Participants were questioned regarding their past and current smoking status. Smoking burden was estimated based on smoking duration, daily exposure, and cumulative smoking exposure burden (CSEB).

### 2.3. Study Procedure

On the day of the study, participants were instructed to refrain from smoking and drinking alcohol or beverages containing caffeine, and to refrain from physical activity for at least 3 h before the electrocardiogram. The tests were performed between 8:00 AM and 1:00 PM to avoid the influence of circadian rhythm on heart rate and ANS function. After resting in a supine position for at least 5 min, ECG electrodes were placed according to standard procedure. Heart electrical activity was recorded using a high-resolution 1200-HR ECG device (Norav Medical, Yokne’am, Israel) at a sampling rate of 2000 Hz. Interbeat (RR) intervals were measured between consecutive beats. Standard deviation of RR interval (SDNN), root mean square of the successive differences (RMSSD), number of intervals, and percentage of adjacent Normal to Normal (NN) intervals that differ from each other by more than 50 ms (NN50 and pNN50, respectively), as well as HRV triangular index (HRVTI) were calculated to quantify the HRV time. Power spectral analysis was conducted using the nonparametric fast Fourier transform. Integrals of the area beneath the power spectral density curve for frequency range were calculated in absolute values of power (ms^2^). The spectral components were divided into very low frequency (VLF, 0.003–0.04 Hz), low frequency (LF, 0.04–0.15 Hz), and high frequency (HF, 0.15–0.4 Hz). The baseline HRV results were also converted to normalized units (n.u.) in accordance with accepted methods [17]. Recordings with inadequate quality and those containing premature ventricular or atrial contractions were repeated. In addition, the natural logarithm (ln) of HRV parameters were calculated.

Data were saved in binary format and processed with a commercial computer software (Norav Medical, version 5.514, Yokne’am, Israel), which has been validated and tested for reproducibility according to accepted standards [18].

Thereafter, an additional 1 min ECG was conducted with the same equipment, during which subjects were asked to remain supine and to breathe deeply in and out six times. Maximum heart rate during expiration (E) and minimum HR during inspiration (I) were measured, and the difference between the two calculated (ΔE/I = maximum HR − minimum HR). The standard deviation of the RR interval and RMSSD were computed for the deep breathing period. The E/I ratio was calculated by dividing the longest mean RR interval during expiration by the shortest RR interval during inspiration [19].

Blood pressure was measured twice with an automated commercial sphygmomanometer (Welch Allyn 4200B-E1), and values were averaged.

### 2.4. Statistical Analysis

Data were analyzed using JMP version 7.0 (SAS Institute, Cary, NC, USA) and MelCalc version 19.1.5 (MedCalc Software bvba, Ostend, Belgium). Continuous variables are expressed as mean and standard error of mean for each parameter. Distribution was evaluated with the Shapiro–Wilk test. Independent-samples Student’s *t*-test or the non-parametric Wilcoxon Rank Sum test was performed for normally distributed and non-normally distributed parameters, respectively. *p* < 0.05 was considered statistically significant. Regression analyses were estimated according to proportion of the variance in the dependent variable that was predictable from the independent variable, between smoking and HRV parameters. The strength of the association was evaluated with Pearson’s correlation coefficient (PCC; r). A mean PCC value of 0.9–1.0 was considered a very strong correlation, a value within the range of 0.7–0.9 was considered a strong correlation, and PCC values within the range of 0.5–0.7 were considered moderate correlations. Categorical outcomes were checked with Chi-square test and are expressed as percentages.

## 3. Results

A total of 242 volunteers were included in the study. Forty-six were active cigarette smokers (19% of the cohort) and 196 were not active smokers (26 were past smokers). The clinical and demographic characteristics of the study groups are outlined in Table 1.

The cohort included 104 men and 138 women. Forty-one smokers provided detailed information about their smoking habits. In the remaining five, smoking exposure was intermittent or could not be clearly determined due to high variability in smoking habits over time. The mean smoking duration was 8.0 ± 1.0 years, with CSEB of 5.3 ± 1.3 pack-years.

Comparative analysis of HRV parameters according to smoking status is presented in Table 2.

ln(SDNN), ln(RMSSD), ln(HRVTI), ln(LF/HF), and LF were normally distributed. There were no significant differences in any of the HRV parameters between groups. Accordingly, regardless of their smoking status, participants had statistically similar mean values of maximum, minimum, and average RR values, as well as similar values of SDNN, RMSSD, HRVTI, NN50, pNN50, VLF, LF, HF, LF/HF ratio, and total power. In addition, there were no significant differences in any of the parameters following logarithmic transformation (Table 2).

Table 3 presents comparative analysis of DBT parameters according to smoking status.

SDNN, RMSSD, VLF, and HF showed a tendency towards lower values in the smokers’ group, as compared with the control group, yet this effect did not reach statistical significance. There were no significant differences between the control and smokers’ groups in ΔE/I, E/I ratio and total power.

Those who were previous smokers had a CSEB of 5.9 ± 2.0 pack/years. A sub-analysis that excluded former smokers and compared active smokers and participants with no history of smoking revealed similar results for all tested parameters.

Correlations between smoking exposure and HRV and DBT parameters are outlined in Table 4 and Table 5, respectively.

A significant correlation (*p* < 0.05) was found between smoking duration and LF, ln(NN50), ln(HF), and ln(LF/HF), but associations were low for all parameters (e.g., r < 0.5). A moderate correlation (r = 0.50) was found between smoking duration and LF/HF ratio. A significant association was found between daily smoking exposure and several HRV parameters including SDNN, RMSSD, HRVTI, NN50, pNN50, LF, LF/HR, ln(SDNN), ln(RMSSD), ln(HRVTI), ln(NN50), ln(pNN50), ln(LF), and ln(LF/HF). All associations were low, other than LF/HR (r = 0.50) and ln(HRVTI) (r = 0.51) in which the association was moderate.

Significant associations were found between CSEB and SDNN, HRVTI, NN50, pNN50, LF, HF, LF/HF, ln(SDNN), ln(RMSSD), ln(HRVTI), ln(NN50), ln(pNN50), ln(LF), ln(HF), and ln(LF/HF). A borderline significance was found between CSEB and RMSSD (p = 0.051). A moderate strength of association was found between CSEB and LF (r = 0.51), LF/HF (r = 0.69), ln(HRVTI) (r = 0.50), and ln(LF/HF) (r = 0.50). The linear regression slopes (β1) were negative for the correlations of SBEB and SDNN, RMSSD, HRVTI, NN50, pNN50, HF, ln(SDNN), ln(RMSSD), ln(HRVTI), ln(NN50), ln(pNN50), ln(HF), and positive for LF, LF/HF, ln(LF), and ln(LF/HF).

Regression analysis between smoking burden and DBT parameters was conducted, and no significant correlations were found. Correlations of borderline significance were found between smoking duration and total power, and between daily exposure and HF. A borderline correlation was also found between CSEB and HF. All associations were low order of magnitude (e.g., r < 0.5).

## 4. Discussion

The influence of active smoking on HRV has been studied by several research groups with limited numbers of participants, and most with no exclusion of patients with chronic diseases [10]. The first published evidence of the association between smoking and ANS dysfunction was published by Penny and Mir in 1986 [20]. They reported decreased HRV parameters in a small cohort of 12 patients. In the following years, several epidemiological studies were conducted in an attempt to shed further light on these associations. Some studies failed to find a relationship between chronic active smoking and decreased HRV [10,13,14], while other studies reported significant associations [7,11,12]. Importantly, few studies attempted to differentiate between the immediate short-term and the cumulative effects of cigarette smoking. In 1990, Hayano [7] and colleagues reported that smoking causes an acute and transient decrease in vagal cardiac control. In 2005, Kobayashi [21] found that LF/HF is significantly increased immediately after smoking. Since these influences may have affected the results of some earlier studies that included people who smoked shortly before the autonomic test, several other papers have attempted to quantify ANS in smokers after a few hours of abstinence [22,23]. Yet, these studies yielded conflicting results and were difficult to interpret in light of comorbidities [22]. For example, in 2019, Murgia and colleagues [15] found a significant relationship between decreased HRV parameters (SDNN and TP) with every 5 pack-year increase. In addition, this study included participants with histories of diabetes, cardiovascular events, and hypertension. Notably, additional autonomic markers were not investigated in any of the previous studies on ANS function among smokers.

In contrast to previous studies, the current study was conducted under strict test conditions and excluded individuals with known chronic diseases to avoid their effects on HRV and DBT. Moreover, participants were instructed to refrain from smoking, caffeine, alcohol, and physical exercise for at least 3 h before the test, thus minimizing the short-term effects of cigarette smoking and other exposures on the study results. We examined only young, apparently healthy adults with no history of chronic diseases and no use of medication.

Smoking prevalence was 19%, which is in line with the smoking rate reported in the general population worldwide (20.5%) [24]. First, we showed that there were no significant differences between the control and smokers’ groups in HRV and heart rate response to DBT. Consistent with our results, Murata [13] and Kageyama [14] did not find a link between current smoking status and HRV.

Second, we found a significant association between smoking burden parameters (smoking duration, daily exposure, CSEB) and several HRV parameters, but associations were low or moderate for all (e.g., r < 0.7). The linear regression slopes (β1) were negative for SDNN, RMSSD, HRVTI, NN50, pNN50, HF, ln(SDNN), ln(RMSSD), ln(HRVTI), ln(NN50), ln(pNN50), ln(HF), and positive for LF, LF/HF, ln(LF), and ln(LF/HF). These changes are suggestive of increased sympathetic nervous system activity. The results are in line with those of Murgia [22] who also observed decreased HRV, with a stronger correlation between SDNN and TP with every 5 pack-year increase and dose-response reductions in SDNN, RMSSD, TP, LF, and HF.

The current study had several possible limitations, including the 5 min HRV test, which might not represent 24 h ANS functions and the influence of the circadian system on autonomous modulation [25,26]. Yet, all participants underwent ECG at about the same time in the morning under standardized conditions in order to minimize the effects of external influences on the results. In addition, it was found that with short-term recordings of less than three minutes, as was done for DBT analysis, frequency domain indexes should be considered more reliable, while time domain indices are preferred with long-term recordings [18].

Another limitation is related to the smoking questionnaires, which did not differentiate between day- and night-time smoking. Night-time smoking in male workers was suggested to affect cardiac autonomic function more strongly and acutely than daytime smoking [21]. Therefore, it remains to be explored whether ANS function is more severely affected in individuals who are accustomed to smoking during the night and not during the day.

Notably, in this study, we included patients with no known cardiovascular disease or risk factors other than family history of diabetes, hypertension, or mild dyslipidemia. Yet, we cannot exclude the possibility that some of our patients had a subclinical cardiac disorder. Although the familial prevalence of hypertension and diabetes was balanced among groups, it remains unknown whether genetic variability might have affected the study outcomes. In addition, the level of physical activity was not controlled. Yet, the negative results of the current study suggest that such a difference, if present, did not cause a significant alteration in ANS activity.

None of our patients engaged in physical activity or drank caffeinated or alcoholic beverages at least 3 h before the study. Yet, we cannot predict whether a longer abstinence would have altered the results.

## 5. Conclusions

Among healthy volunteers with no known cardiovascular disease, who smoked for a short duration, there were no significant alterations in ANS function. Yet, a significant association between parameters of smoking exposure and a trend towards abnormal HRV values may suggest that a longer duration of smoking will have more substantial adverse effects on the ANS. These associations between smoking exposure and markers of sympathetic overactivity and decreased parasympathetic tone should be regarded with caution because significant associations were of low or moderate strength. Additional studies including patients with a very long smoking exposure and no cardiovascular risk factors should be conducted to verify the extrapolated conclusions suggested in the current research.

## Figures and Tables

**Table 1:** Clinical characteristics of the study participants.

Parameter	Controls (N = 196)	Smokers (N = 46)	*p*-Value
Age (years)	34.1 ± 1.0	30.5 ± 1.5	0.10
Men, n (%)	80 (40.8)	24 (52.2)	0.19
BMI (kg/m^2^)	24.2 ± 0.3	23.9 ± 0.5	0.98
SBP at rest (mmHg)	117 ± 1	117 ± 2	0.88
DBP at rest (mmHg)	74 ± 1	74 ± 2	0.76
Diabetes mellitus, n (%)	0 (0.0)	1 (0.5)	1.00
Hypertension, n (%)	0 (0.0)	0 (0.0)	1.00
Mild dyslipidemia, n (%)	26 (13.3)	6 (13)	1.00
Chronic medication use, n (%)	0 (0)	0 (0)	1.00
Fx hypertension, n (%)	78 (39.8)	25 (54.4)	0.09
Fx diabetes, n (%)	87 (44.4)	17 (37.0)	0.41
Fx heart diseases, n (%)	91 (46.4)	20 (43.5)	0.75

BP = blood pressure, SBP = systolic BP, DBP = diastolic BP, NS = non-significant, BMI = body–mass index. Fx = family history in a first degree relative, at any age.

**Table 2:** Values of HRV parameters in smokers and nonsmokers.

Parameter	Controls (N = 196)	Smokers (N = 46)	*p*-Value
Maximum RR (ms)	1105.5 ± 13.0	1099.8 ± 27.7	0.95
Minimum RR (ms)	734.5 ± 8.5	724.1 ± 14.3	0.90
Average RR (ms)	922.7 ± 10.1	913.9 ± 18.1	0.98
SDNN (ms)	603 ± 2.0	61.9 ± 4.2	0.57
RMSSD (ms)	52.4 ± 2.4	57.8 ± 6.7	0.65
HRVTI	16.9 ± 0.5	17.6 ± 1.0	0.43
NN50	36.7 ± 2.2	42.6 ± 5.0	0.38
pNN50	11.9 ± 0.7	13.5 ± 1.7	0.50
VLF (ms^2^)	188.3± 6.0	183.0 ± 16.0	0.35
LF (ms^2^)	366.9 ± 7.0	389.1 ± 14.4	0.92
HF (ms^2^)	160.5 ± 6.2	158.1 ± 13.5	0.82
LF/HF	1.5 ± 0.1	1.7 ± 0.3	0.84
LF (n.u.)	45.1 ± 1.2	44.8 ± 2.8	0.89
HF (n.u.)	42.6 ± 1.3	42.5 ± 3.1	0.96
Total power (ms^2^)	554.4 ± 5.8	551.7 ± 15.2	0.70
Ln(SDNN)	4.0 ± 0.0	4.0 ± 0.1	0.61
Ln(RMSSD)	3.8 ± 0.0	3.8 ± 0.1	0.62
Ln(HRVTI)	2.8 ± 0.0	2.8 ± 0.1	0.77
Ln(NN50)	3.1 ± 0.1	3.1 ± 0.2	0.47
Ln(pNN50)	1.9 ± 0.1	1.9 ± 0.2	0.63
Ln(VLF)	5.1 ± 0.0	5.0 ± 0.1	0.35
Ln(LF)	5.0 ± 0.0	5.0 ± 0.1	0.99
Ln(HF)	4.9 ± 0.0	4.7 ± 0.1	0.82
Ln(LF/HF)	0.1 ± 0.1	0.1 ± 0.1	0.59
Ln(total power)	6.3 ± 0.0	6.3 ± 0.0	0.70

RR = interbeat; SDNN = standard deviation of the RR interval; RMSSD = square root of the mean squared differences of successive RR intervals; HRV = heart rate variability; HRVTI = HRV triangular index; NN50 = number of Normal to Normal (NN) intervals differing by >50 ms from the preceding interval; pNN50 = NN50 divided by the total number of RR intervals; VLF = very low frequency; LF = low frequency; HF = high frequency; n.u. = normalized units.

**Table 3:** Values of deep breathing test (DBT) characteristics in smokers and nonsmokers.

Parameter	Controls (N = 196)	Smokers (N = 46)	*p*-Value
Maximal RR (ms)	1142.0 ± 16.0	1128.4 ± 23.6	1.00
Minimal RR (ms)	673.0 ± 7.4	667.4 ± 11.8	0.92
SDNN (ms)	129.4 ± 8.1	116.0 ± 5.3	0.82
ΔE/I (bpm)	37.2 ± 1.1	37.0 ± 1.9	0.88
E/I ratio	1.7 ± 0.0	1.7 ± 0.1	0.93
RMSSD (ms)	88.9 ± 4.0	82.4 ± 5.5	0.85
VLF (ms^2^)	98.2 ± 5.9	92.5 ± 10.1	0.97
LF (ms^2^)	366.9 ± 7.0	389.1 ± 13.9	0.16
HF (ms^2^)	108.4 ± 4.4	94.8 ± 7.6	0.25
Total power (ms^2^)	582.8 ± 5.9	586.6 ± 10.4	0.61

Non-significant *p*-value > 0.05; RR = interbeat; SDNN = standard deviation of the RR interval; RMSSD = square root of the mean squared differences of successive RR intervals; HRV = heart rate variability; NN50 = number of Normal to Normal (NN) intervals differing by >50 ms from the preceding interval; pNN50 = NN50 divided by the total number of RR intervals; VLF = very low frequency; LF = low frequency; HF = high frequency; bpm = beats per minute.

**Table 4:** Regression analysis between smoking burden and HRV parameters.

	Smoking Duration (Years)			Daily Exposure (Packs/Day)			Cumulative Smoking Exposure Burden (Pack-Years)		
Parameter	β1	r	*p*-Value	β1	r	*p*-Value	β1	r	*p*-Value
Maximal RR (ms)	−1.04	0.00	0.83	−86.36	0.26	0.10	−5.79	0.26	0.10
Minimal RR (ms)	1.28	0.10	0.62	17.54	0.10	0.56	0.66	0.00	0.74
Average RR (ms)	0.71	0.00	0.83	−28.13	0.14	0.44	−2.23	0.14	0.36
SDNN (ms)	0.04	0.00	0.96	−20.93	0.41	0.01	−1.18	0.35	0.03
RMSSD (ms)	0.21	0.00	0.86	−27.58	0.35	0.04	−1.73	0.32	0.05
HRVTI	−0.19	0.20	0.23	−5.35	0.48	0.00	−0.33	0.45	0.00
NN50	−1.01	0.20	0.23	−21.72	0.36	0.02	−1.64	0.41	0.01
pNN50	−0.24	0.14	0.41	−7.22	0.35	0.02	−0.51	0.37	0.02
VLF (ms^2^)	−2.07	0.14	0.45	−2.78	0.00	0.93	0.52	0.00	0.80
LF (ms^2^)	5.28	0.41	0.01	64.51	0.44	0.00	4.99	0.51	0.00
HF (ms^2^)	−4.13	0.28	0.08	−32.90	0.20	0.22	−3.83	0.35	0.03
LF/HF	0.25	0.50	0.00	1.59	0.50	0.00	1.46	0.69	0.00
Total power (ms^2^)	−1.83	0.10	0.50	23.52	0.10	0.45	2.04	0.17	0.33
Ln(SDNN)	−0.01	0.10	0.55	−0.35	0.45	0.00	−0.02	0.41	0.01
Ln(RMSSD)	−0.02	0.17	0.30	−0.52	0.40	0.01	−0.04	0.46	0.00
Ln(HRVTI)	−0.01	0.24	0.11	−0.32	0.51	0.00	−0.02	0.50	0.00
Ln(NN50)	−0.07	0.32	0.04	−0.87	0.35	0.03	−0.08	0.49	0.00
Ln(pNN50)	−0.06	0.28	0.08	−0.90	0.35	0.03	−0.08	0.47	0.00
Ln(VLF)	−0.02	0.17	0.28	0.09	0.10	0.66	0.01	0.10	0.55
Ln(LF)	0.02	0.20	0.20	0.39	0.35	0.03	0.03	0.36	0.02
Ln(HF)	−0.04	0.35	0.04	−0.31	0.24	0.15	−0.04	0.41	0.01
Ln(LF/HF)	0.06	0.35	0.03	0.69	0.37	0.02	0.06	0.50	0.00
Ln(total power)	−0.01	0.14	0.37	0.07	0.14	0.38	0.01	0.14	0.37

RR = interbeat; SDNN = standard deviation of the RR interval; RMSSD = square root of the mean squared differences of successive RR intervals; HRV = heart rate variability; HRVTI = HRV triangular index; NN50 = number of Normal to Normal (NN) intervals differing by >50 ms from the preceding interval; pNN50 = NN50 divided by the total number of RR intervals; VLF = very low frequency; LF = low frequency; HF = high frequency.

**Table 5:** Regression analysis between smoking burden and DBT parameters.

	Smoking Duration (Years)			Daily Exposure (Packs/Day)			Cumulative Smoking Exposure Burden (Pack-Years)		
Parameter	β1	r	*p*-Value	β1	r	*p*-Value	β1	r	*p*-Value
Maximum RR (ms)	1.18	0.00	0.78	−17.56	0.00	0.72	−1.55	0.10	0.63
Minimum RR (ms)	2.32	0.17	0.26	9.41	0.00	0.69	1.16	0.10	0.46
SDNN (ms)	−0.88	0.14	0.35	−12.07	0.17	0.26	−1.07	0.24	0.14
ΔE/I (bpm)	−0.17	0.10	0.61	−2.14	0.10	0.58	−0.23	0.14	0.37
E/I ratio	−0.00	0.00	0.85	−0.05	0.10	0.58	−0.01	0.14	0.43
RMSSD (ms)	0.03	0.00	0.97	−12.10	0.17	0.28	−0.91	0.20	0.22
VLF (ms^2^)	−2.40	0.22	0.18	10.04	0.14	0.36	0.53	0.00	0.70
LF (ms^2^)	0.10	0.00	0.97	6.74	0.00	0.81	0.84	0.10	0.66
HF (ms^2^)	0.01	0.10	0.57	−26.65	0.28	0.08	−1.86	0.30	0.06
Total power (ms^2^)	−3.35	0.28	0.07	3.88	0.00	0.86	−0.33	0.00	0.82

RR = interbeat; SDNN = standard deviation of the RR interval; RMSSD = square root of the mean squared differences of successive RR intervals; HRV = heart rate variability; HRVTI = HRV triangular index; NN50 = number of Normal to Normal (NN) intervals differing by >50 ms from the preceding interval; pNN50 = NN50 divided by the total number of RR intervals; VLF = very low frequency; LF = low frequency; HF = high frequency; bpm = beats per minute.
